# Need for accessible infertility care in Ghana: the patients’ voice

**Published:** 2016-06-27

**Authors:** Nana Yaw Osei

**Affiliations:** CEO of the Association of childless couples of Ghana (ACCOG), Accra, Ghana.

**Keywords:** Affordable IVF, childlessness, Ghana, infertility, sociocultural consequences

## Abstract

According to the Ghana Statistical Service (GSS) infertility and childlessness are the most important reason for divorce in Ghana. The traditional Ghanaian society is pro-natal and voluntary childlessness is very uncommon. Patient groups are almost non-existent in Sub-Saharan Africa, aggravating the situation of childless couples. Due to the lack of enough and affordable high quality infertility services, many women resort to traditional healing, witchcraft and spiritual mediation. Considering the severe sociocultural and economic consequences of childlessness, especially for women, there is an urgent need for accessible and affordable high quality infertility care in Ghana.

## Introduction

The list of Reproductive health care services as contained in the Programme of Action of the International Conference on Population and Development (ICPD) held in Cairo, Egypt, in September 1994 includes the “prevention and appropriate treatment of infertility”. Unfortunately, this is an area that has been overlooked so far. While the health insurance systems of most developed nations take care of ARTs treatments and in some instances people going through the treatment receive refunds for as much as six cycles, their colleagues in developing countries including Ghana are left to their fate. It is very disheartening that infertility and people living with infertility in developing nations particularly Africa, do not have the needed support. Africa is said to be poor, overpopulated, with high fertility rates etc. Consequently it is difficult to get funding/support for infertility activities even though people living with infertility in Africa are the worse hit among infertility patients worldwide.

## Infertility in Ghana

The traditional Ghanaian society is pro-natal, where the ultimate purpose of marriage is to produce children who will continue the name of the family (Gyekye, 1996; Nukunya, 2003). The joy of couples is to have children after marriage since ‘voluntary childlessness’ cannot be found in the dictionary of people in Ghana. According to Larsen (2000) the primary infertility rate for women in Ghana is estimated to be 2%, the secondary infertility rate was 14%.

Information on male infertility in Ghana is very scanty except that in a reproductive health report to the World Health Organisation in the year 2003, the Ghana Health Service (GHS) reported that male infertility was emerging in the Upper East Region of the country.

In Ghana, a number of studies have reported psychological distress among infertile women. Infertility-related stress and stigma were found among women seeking infertility treatment in Southern Ghana (Donkor and Sandall, 2007; 2009). The authors reported that 23% of the women experienced moderate stigma and 41% experienced severe infertility-related perceived stigma. Women who reported severe levels of perceived stigma had the highest mean score for fertility-related stress. Furthermore, two recent studies in Ghana reported on the mental health effects of infertility among Ghanaian women (Fledderjohann, 2012; Naab et al., 2013). According to these studies infertile Ghanaian women experience many psychosocial consequences of childlessness such as social stigma, marital instability and mental health problems including worrying, crying for long periods, and insomnia (Fledderjohann, 2012). Similarly, 53% of women seeking treatment for fertility problems in Ghana were depressed (Naab et al., 2013). Some individuals facing infertility who cannot withstand the high stigma on childlessness in Ghana also end up taking their own life.

This confirms previous statements that the consequences of involuntary childlessness are usually more dramatic in developing countries when compared to Western societies, particularly for women (Dyer et al., 2004; 2005; Ombelet et al., 2008; Van Balen and Bos, 2009).

## Treatment seeking behaviour of childless couples in Ghana

In Ghana, apart from biomedical causes of infertility, traditional or religious causes of infertility such as spiritual and witchcraft have been cited (Donkor and Sandall, 2009). Consequently, in the treatment of infertility, many women resort to traditional healing and spiritual mediation (including churches) as well as orthodox biomedicine. Some women are deeply convinced of supernatural causes, and so they patronize the services of traditional and religious healers for spiritual redress. At present it is very common in Ghana to see some women testifying in churches to the fact that they have been able to conceive and given birth through prayers. Women therefore form the majority of attendants at prayer camps and other alternative health care sources in Ghana’s pluralistic health context (Sackey, 1999).

Even when medical means are used, it is mostly the traditional healers that get a lot of patronage in view of certain practices in the country and the perception that their services are cheaper. The irony of the situation is that hospitals are not allowed to advertise themselves in the media but traditional healers and churches are free to do any adverts in any media and so they make all sorts of claims regarding their ability to treat infertility. The resort to spiritual and traditional healing in the end worsen the plight of many people living with infertility since they waste a lot of time doing these and in the end may have to rely on donor gametes for conception which increase the cost of treatments.

In the face of all these, a question that one can pose is “what support systems are there in place for childless persons, particularly women, in Ghana?” In the developed countries there are support groups, infertile couples receive professional information and counselling. However, in a strong pro-natal society like Ghana such support groups are non- existent. This situation can aggravate the situation of childless couples and individuals living with infertility in Africa.

It is therefore of uttermost importance that counselling and public education are integrated seriously in infertility care in Africa and this can be best done if patients organisations are involved.

## Should the focus be on women in Africa?

It is true that women are the highest hit when it comes to dealing with issues relating to infertility and so they are used mostly in advocacy and are often seen telling their stories. A typical African man irrespective of level of education hardly believes he can be faced with infertility and so use every possible means, whether good or bad, to get a child in an effort to “prevent a disgrace”. The belief that men cannot be faced by infertility is generally accepted by many societies in Africa including Ghana. I believe the focus should be on the men more than the women in Africa in our advocacy and public education work. Once men understand infertility situations, they’ll pull the women along and they’ll all be happy people and children will be secondary issue.

## Need for accessible infertility care in Ghana

Financial constraint has been seen as the key factor preventing infertile couples from accessing the treatment they need. Numerous people living with infertility in Ghana who now look to us for hope because we have made them aware about the emergence of an affordable fertility treatment through The Walking Egg project (Ombelet, 2013; 2014; Van Blerkom et al., 2014).

According to the Ghana Statistical Service (GSS) infertility and childlessness are probably the most important reasons for divorce in Ghana. Accessible infertility care can therefore help to reduce if not eliminate childlessness related divorce and polygamy in Ghana. Accessible infertility care can help reduce this.

Infertility brings domestic violence among couples and extended family relations. Accessible infertility care can help reduce this.

Infertility sometimes makes people poor because people sell their properties and other belongings to search for a child. Accessible infertility care can help reduce this.

The high stigma on childlessness in Ghana brings psychological problems to many couples and individuals facing infertility and this make some people commit suicide sometimes. Accessible infertility care can help reduce this.

## Conclusion

Due to the severe socio-cultural and economic consequences of childlessness and infertility in Ghana, there is an urgent need for accessible and affordable high quality infertility care.
